# Cartography of the lines of force that run through rural nurses’ work processes

**DOI:** 10.1590/0034-7167-2025-0060

**Published:** 2026-07-17

**Authors:** Bruno Neves da Silva, Nilba Lima de Souza, Deise Lisboa Riquinho, Cristina Andrade Sampaio, Gerlane Cristinne Bertino Veras, Valéria Gomes Fernandes da Silva, Mariana Castilho Valle, Erika Simone Galvão Pinto

**Affiliations:** I Universidade Federal do Rio Grande do Norte. Natal, Rio Grande do Norte, Brazil; II Universidade Federal do Rio Grande do Sul. Porto Alegre, Rio Grande do Sul, Brazil; III Universidade Estadual de Montes Claros. Montes Claros, Minas Gerais, Brazil; IV Universidade Federal de Campina Grande. Cajazeiras, Paraíba, Brazil

**Keywords:** Rural Nursing, Primary Health Care, Rural Health, Nursing Care, Qualitative Research., Enfermería Rural, Atención Primaria de Salud, Salud Rural, Atención de Enfermería, Investigación Cualitativa.

## Abstract

**Objectives:**

to map the lines of force that run through rural nurses’ work processes.

**Methods:**

a qualitative study, based on the Deleuze and Guattari cartography framework. Data were collected through discursive interviews and the instruction to the double technique, conducted between November-December 2022 and September 2024, with eight nurses working in the Paraíba hinterland, Brazil. Foucault’s discourse analysis was carried out.

**Results:**

limiting rigid lines were detected in professional practices, such as fixed agendas, top-down campaigns, and comparisons with urban settings. Conversely, lines of flight were observed, manifested in the strengthening of ties with the community, promoting inventive practices and adaptations in care.

**Final Considerations:**

the findings broaden the science of nursing by making practices in rural territories visible, offering analytical support for understanding local challenges and unique characteristics. The formulation of specific policies, continuing education, and the valorization of rural nursing as a strategic field for care are recommended.

## RESUMEN

**Objetivos:**

mapear las líneas de fuerza que atraviesan los procesos laborales de enfermeras rurales

**Métodos:**

estudio cualitativo basado en el marco cartográfico de Deleuze y Guattari. Los datos se recopilaron mediante entrevistas discursivas y la técnica de la doble instrucción, realizadas entre noviembre-diciembre de 2022 y septiembre de 2024, con ocho enfermeras que trabajan en el interior de Paraíba, Brasil. Se llevó a cabo el análisis del discurso de Foucault.

**Resultados:**

e detectaron limitaciones rígidas en las prácticas profesionales, como agendas fijas, campañas verticales y comparaciones con entornos urbanos. Pero, se observaron vías de escape, manifestadas en el fortalecimiento de los vínculos con la comunidad, la promoción de prácticas innovadoras y adaptaciones en la atención.

**Consideraciones Finales:**

los hallazgos amplían la ciencia de la enfermería al visibilizar las prácticas en territorios rurales, ofreciendo apoyo analítico para comprender los desafíos locales y sus características únicas. Se recomienda la formulación de políticas específicas, formación continua y valoración de la enfermería rural como un campo estratégico para la atención.

## INTRODUCTION

Brazil’s rural population is characterized by diversity in ethnicity, people, race, religion, economics, society, production systems, technology, ecosystems, and vast, rich biodiversity. Consequently, the richness of rural Brazil extends beyond natural resources to the diversity of its people^(1)^.

For this reason, it should be understood in terms of its social, political, economic, and productive interrelationships, rather than merely as a population inhabiting a non-urbanized area. The rural territory encompasses agriculture, ecological sustainability, and the organization of social groups, rural workers, and their families, among other functions^(2)^.

Healthcare in rural areas is related to regional, demographic, and economic factors that influence the planning and management of healthcare services^(3)^. The rural population is particularly vulnerable to health problems due to a lack of primary care, inadequate sanitation, and difficulty coordinating services. This highlights the importance of implementing public policies in rural areas^(4)^. From this perspective, public health policies for these populations must be more closely aligned with the health situation in each territory^(2)^.

The persistence of inequalities in healthcare processes in rural areas indicates a discrepancy between current practices and the expectations of Primary Health Care (PHC), which is often the only available health resource and is directly integrated into the Brazilian rural context^(5)^. This approach involves developing strategies that promote individual health, prevent disease, and address the real and potential needs and demands of the rural population^(6)^.

The PHC in Brazil’s Unified Health System (In Portuguese, *Sistema Único de Saúde* - SUS) has been implemented through the Family Health Strategy (FHS). Although FHS performs worse in rural areas, it has represented progress in terms of population coverage. FHS is a counter-hegemonic proposal to the predominant Flexnerian biomedical model in Brazil^(7)^. In this scenario, nurses take a prominent role as a coordinator of the actions developed by FHS, which contributes to broadening the horizons of their professional practice^(8)^.

Although there is no consensus on the definition of rural nursing, authors generally consider it to be the provision of healthcare by nurses to people living in sparsely populated areas^(9)^. The practice of rural nursing should demonstrate a commitment to solving problems in diverse contexts, building different forms of care, and critically reflecting on actions performed^(10)^.

In rural settings, nurses’ work process demands specialized care due to the environment’s unique characteristics. These include differentiated and dynamic geographical territories, as well as specific health and disease processes, such as the persistence of endemic patterns and low quality of life. This requires the implementation of measures to overcome recurring healthcare problems^(11,12)^. Despite these characteristics, rural nurses’ work is an effective way to reduce health disparities^(13-15)^.

Although they are integrated into PHC and are primarily responsible for coordinating care and managing Family Health Units, improving nurses’ practices contributes to the advancement of PHC. This reinforces the idea that strengthening PHC is key to combating inequities in rural areas^(5)^. From this perspective, the relevance of this study is clear. It allows us to reflect on and improve the driving forces behind rural nurses’ work processes. This involves recognizing the factors that influence their performance. The goal is to transform the healthcare reality, promote the comprehensive health of the rural population, and improve their quality of life and well-being.

To achieve this objective, we adopted cartography as the conceptual framework of this study, as proposed by Deleuze and Guattari. This approach enables us to examine the evolving processes of professional practice and map the lines of force, tensions, repetitions, and innovations that comprise daily healthcare work. This epistemic approach is still under-explored in the national context and absent from national and international debates on nursing in rural areas. Without exhausting the subject, this study opens the door to other ways of understanding rural nurses’ professional practices, taking into account the unique aspects of care and the forces that influence it. In light of this, the study sought to answer the following theoretical question: How do lines of force run through the organization of rural nurses’ work processes?

## OBJECTIVES

To map the driving forces that run through rural nurses’ work processes.

## METHODS

### Ethical aspects

This study derives from a doctoral thesis entitled “Micropolitics of the work process of rural nurses: a cartography of care practices” (“*Micropolítica dos processos de trabalho de enfermeiras rurais: uma cartografia das práticas de cuidado*”). It was approved by the *Universidade Federal do Rio Grande do Norte* Research Ethics Committee, under Opinion 5,695,462. The research was guided by Resolutions 466/2012 and 510/2016 of the Brazilian National Health Council. Study participants’ consent was obtained by explaining the objectives and having them read and sign the Informed Consent Form in duplicate. Anonymity of research participants was guaranteed, and the codename “NUR” followed by a cardinal number was used for the use of their statements throughout the text, according to the order in which each data production procedure was carried out.

### Theoretical-methodological framework

This study is based on the cartography of Gilles Deleuze and Felix Guattari. Cartography is linked to post-structuralist thought and represents a contemporary methodological approach. Depending on the application, the researcher’s intentions, and the role it plays in the research process, it can be interpreted as methodology, method, or methodological procedure^(16)^.

In Deleuze and Guattari, the term “cartography” suggests that the meshes that compose a rhizome do not have depths to be scrutinized. Rather, they are engendered in lines that intertwine to construct plots and compose diverse trajectories forged in distinct intensities (political, linguistic, aesthetic, economic, and biological, among others). These trajectories indicate movements that constitute a map of intensities and affects in the formation of existential territories^(17)^.

Authors^(18)^ highlight that the study of these lines is important for understanding subjectivation processes. These are the relationships of meaning forces that subjects in society and the world weave. These lines often escape the subjects themselves and delineate other experiences of meaning or make existing ones chaotic.

Large strata or molar sets (social classes, genders, family-school paths, school-work paths, etc.) are characterized by lines of hard segmentation. These lines are rigid and related to the formation of subjects. These lines outline classifications, such as class and level, and function dichotomously. They allude to aspects of reality that appear to be natural, established, and permanent. These aspects tend to ward off criticism and questioning^(18,19)^.

In their context, lines of flight constitute ruptures that undo the “self” and its established relationships, leading to the pure experimentation of becoming, albeit momentarily. Often, lines of flight must be invented without a guiding model. They represent the lines that liberate desire from imprisonment in strata, so they must be produced in the cartographies of subjects, especially when their potential is threatened by accentuated stratifications^(18)^.

These lines allow for flight and resistance to the established order. They define multiplicities and highlight the reality of a finite number of dimensions that are filled by this^(20)^.

### Study design

This is a qualitative study. The Standards for Reporting Qualitative Research were used as a guide to prepare the research report, as required by the journal.

### Methodological procedures

Nurses with one or more years of experience in rural areas were included in the study, a period deemed necessary for these professionals to develop their identity as rural nurses. Professionals who were absent from work during the data collection period due to leave, vacation, absence, or other reasons were excluded. Following the selection criteria, participants were contacted in-person using theoretical sampling.

### Study setting

The study was conducted in FHS rural units located in the 10^th^ Health Region of Paraíba, Brazil.

### Data collection and organization

Data were collected from selected interviewees between November 2022 and September 2024. A nurse researcher with a master’s degree conducted the process. This researcher was familiar with the adopted framework and experienced in qualitative studies. The interviews took place in the nurses’ offices in FHS units. The nurses indicated this location due to its easy access and because it represented a suitable, private environment for the occasion.

Data were produced through discursive interviews. A script with open-ended questions guided the process. Rather than acting as a normative instrument, the script served as a performative operator, capable of activating meanings in flux within the fabric of the interview. To complement the interviews, the technique of instructing the double was used. This technique consisted of giving the following command to the interlocutors, “Imagine that I am your double, and that tomorrow, I will replace you at work. What instructions would you give me so that no one notices the change?”.

This technique originates from psychology and aligns with the intentions of this study. It is particularly well-suited to cartography because it fosters an embodied, situated, and reflective discourse that enhances the emergence of affects, gestures, and knowledge permeating practices. Using this technique broadened the analytical scope, producing a narrative shift that provided access to the singularities of the work beyond conventional statements. This is in line with the cartographic interest in following modes of existence in their processuality.

The criterion for interrupting data production was guided by the recurrence of certain meaningful effects and the perception of forces that constituted an analytical field consistent with the study’s objective and cartographic principles. In cartography, the goal is not to saturate the data since the research does not begin with representation or totality but with monitoring processes in their multiplicity and movement. Furthermore, in light of precepts of discourse analysis, the possibility of achieving full saturation is questionable, since a given discursive order, situated in its time, remains continuously susceptible to (re)interpretations *a posteriori*.

The minimum interview duration was 12 minutes, and the maximum was 34 minutes. Eight interviews were conducted, which were audio-recorded and subsequently transcribed, forming the research *corpus*.

### Data analysis

Data production and analysis were simultaneous, drawing on Michel Foucault’s theories of discourse for analysis. According to the author, discourse analysis does not reveal the universal meaning of words, but rather exposes the web of imposed rarefactions with fundamental affirming power^(21)^. From Foucault’s perspective, analyzing the discursive field involves understanding an utterance in terms of its singularity and situational specificity. It also involves identifying the conditions of its existence, adequately delimiting its contours, establishing its connections with other associated utterances, and demonstrating which forms of enunciation are excluded. The aim is not to seek the silent conversation of a different discourse behind what is explicit. Rather, it is necessary to demonstrate why it could not be otherwise, how it precludes any alternative possibility, and how it occupies a unique place among others that no other could occupy^(22)^.

From this perspective, the analysis identified discursive regularities in the participants’ statements and the truth effects underlying certain approaches to caring for and being a rural nurse. Discursive chunks were constructed in which fragments expressing lines of force, such as norms, ruptures, and resistances in work processes, were grouped. This analytical process was integrated into the cartography framework, enabling the identification of emerging discourses at the micropolitical level and the mapping of their implications for professional practices.

These chunks were not constructed using fixed steps or prior categorization. They were developed through an immanent analysis of the data in accordance with cartography. This process involved monitoring the evolving meanings in encounters between the researcher, discourses, and the field. Attention was given to intensities, repetitions, and variations. The chunks were organized by identifying lines of force that encompassed the work processes. These lines were sometimes normative repetitions (hard lines) and other times inventive variations (lines of flight), which allowed visualizing zones of tension, displacement, or reinvention in the analyzed practices.

## RESULTS

Seven female nurses and one male rural nurse participated in the study after an initial refusal due to lack of time. The participants were between 28 and 56 years old. Seven of the nurses had specializations in various areas of nursing, including PHC, and one had a master’s degree. They had worked in rural areas for between one and 14 years. All participants stated that they had not participated in any specific training to work in rural areas before or after entering the field. The feminine plural was chosen to reflect the predominantly female composition of the study and the historical and sociocultural prevalence of women in nursing.

Regarding the data obtained from the interviews and the technique of instructing the double, the cartography consisted of two discursive chunks. Each statement was analyzed according to its condition of possibility within the power-knowledge regime, as well as its placement within hard lines or lines of flight.

### “There are professionals in rural areas who do not want to get their hands dirty”: the hard lines that run through the micropolitics of rural nurses’ work processes

It was understood that the statements surrounding the discourse of professionals materialized rigid lines of segmentation, stifling care practices. Examples include fixed monthly agendas or schedules without programmed demand and the omission of nursing consultations from the nursing care work process. The analysis revealed that weekly planning functions as a disciplinary technology that fragments time, classifies bodies, and legitimizes a medical perspective. This technology only exists because it is supported by ministerial devices, protocols, and indicators that define “scientifically correct” care. These indicators hinder inventive developments that could respond to the territory’s unique needs. The following discourse illustrates this intersection in micropolitics.

*My work schedule is 40 hours a week, Monday through Friday. On Monday mornings, I provide prenatal care, and in the afternoon, I see walk-in patients. On Tuesday mornings, I conduct rapid tests, and in the afternoon, I see walk-in patients. On Wednesday mornings, I have well-child visits, and in the afternoon, I see walk-in patients. On Thursdays, I have vaccination appointments, and in the afternoon, I do home visits. And on Fridays, I conduct the HIPERDIA program, which is care for hypertensive and diabetic patients. On Friday afternoons, I have a day off.* [...] *In spontaneous demand, issues such as wound care and administering medication arise* [...]. (NUR02)

This framing is reinforced by a campaign-driven model dictated by management that prioritizes actions such as “Pink October” or “Blue November”. This model distances itself from the specific demands of the rural population, such as promoting the health of farmworkers. Although these campaigns benefit the population, they function as rituals of truth that define who should be educated, on what topics, and when and how this should happen. This reinforces rural nurses’ institutional authority.

Topics such as pesticides, worker health, and rural violence are excluded from discussion because they do not align with the surveillance codes that underpin the unit’s funding and objectives. Each campaign takes a hardline approach, creating a network that captures care flows on fixed dates, repeats discourses, and restricts inventive developments that engage with the territory’s specificities. These actions are hindered by inaccessible schedules, predetermined themes, and a numerical logic of service provision, making it difficult to address rural particularities.

[...] *it’s more like a campaign* [the educational practices]*. It’s here in the waiting room, “Pink October”, “Blue November”. Our “Blue November” will be this coming Thursday, the last Thursday of the month* [...] *we’re going to have an educational talk with the physician, and the users who come will have the PSA test requested, and I’ll also be doing the rapid tests.* (NUR01)

Although management dictates the format of educational campaigns, nurses are responsible for updating these models through their passive attitude. Nurses are also responsible for creating their own monthly schedules. As discussed previously, these schedules generally do not incorporate educational practices into daily routines beyond actions linked to campaigns. Furthermore, there is a discourse that blames users for low adherence to health promotion actions while exempting professionals from their share of responsibility.

[...] *to run a campaign, we need to offer something different to get people to come, but they don’t want to waste time.* (NUR01)

[...] *we schedule* [educational activities], *but the people here aren’t really into lectures* [...] *they don’t value that kind of thing.* (NUR05)

However, some argue that the population does value educational initiatives. In some cases, the population has demonstrated significant engagement with activities that align with their capabilities.

*We even organized a “Pink October” event, the first nighttime event, and it was* [...] [gestures with hands]: *we were surprised! Even the* [health] *secretary was surprised by the size of the crowd!* (NUR08)

The population is portrayed as uneducated, and the community’s refusal to participate, as seen in previous speeches, affirms professional authority. However, the counterpoint mentioned in the above speech breaks with this truth, revealing that the absence was a result of imposed formats, not disinterest.

The rural nurse speaks from a position of authority when referring to the population because she is a trained professional who possesses legitimate, socially recognized knowledge. This knowledge gives her power in the relationship that circulates through the discourse. The rural population is described homogeneously and in terms of a lack (“they do not value it”), which reinforces an implicit hierarchical relationship, positioning it as lacking knowledge or interest in what the nurse considers important. Thus, rather than engaging with contextual knowledge and practices, nurses’ discourses suggests that the rural population is expected to adopt the logic of the biomedical model without genuine adaptation to the context.

Furthermore, the absence of opportunities for dialogue and the establishment of community bonds is evident in the discourses and schedules, which include entire shifts of “free demand” that are underutilized for participatory actions. The absence of community meetings reveals a gap. Thus, nurses recognize the absence of social participation, a practice historically relegated to a secondary position within the biomedical system. This signals an enunciative rupture in which care is perceived as being produced without the community’s voice. The mere possibility of meetings makes clear that the dominant narrative depends on the invisibility of others.

[...] *here, for the community, we never schedule a meeting... it’s actually a good thing, isn’t it? To invite the community to participate, but people don’t want to waste time* [...] *unfortunately, that’s the biggest difficulty.* (NUR01)

Furthermore, the researcher-cartographer caused a disruption in the nurse’s discursive practice. This disruption was observed in the phrase “it’s actually a good thing, isn’t it?”. This phrase functioned as a micro-intervention, shifting professionals away from the automatism of the agenda. This disruption confirms the power of cartography as a method of observation and as a device that produces ethical-political movements within the work process of “nursing care” by touching reality.

Rigid patterns were also detected in the patient care flow, which diminishes nurses’ autonomy. Typically, nursing technicians triage users and decide whether referral to nurses, physicians, or dentists is necessary. This triage establishes a hierarchy between knowledge and institutional roles. This organization reinforces the centrality of medical knowledge, relegating nursing consultations to accessory roles. This constitutes a functional redistribution based on medical-biological epistemology.

*The flow is generally this: the patient is attended to at reception, and from there, they are referred to triage; they go through an assessment by a nursing technician, who also assesses the need for care, whether it is urgent or not* [...] *then they are referred to the nurse, the physician, or the dentist.* (NUR02)

Despite their discourse’s implicit critique of biomedicalization, rural nurses adhere to and reproduce protocols because they are considered *sine qua non* for care. In this context, they are not merely technical instruments, but rather, artifacts of normative power that lend legitimacy to professional practice. Questioning these limits carries symbolic and institutional risks, such as disqualification or the imposition of symbolic or practical sanctions by the team or management. Thus, even when they are uncomfortable with the limits imposed by biomedical protocols, nurses often reproduce them because doing so anchors the possibility of technical recognition, institutional belonging, and legal security for their actions.

Thus, constant tension permeates the field of rural nursing. On the one hand, there is the power of situated, affective, and inventive care. On the other hand, there is the need to conform to a normative logic that standardizes bodies, knowledge, and gestures. This ambivalence perpetuates the maintenance of the *status quo*, inhibiting creative or deviant practices and capturing care with indicators, goals, and predefined formats that do not always respond to the singularities of rural life.

The analytical process revealed hard lines from the personal conceptions of professional nurses, as well as comparisons between work processes in rural and urban areas. The statement outlines the boundaries of the sanitary discourse, in which dust, beliefs, and traditions are considered irrational yet tolerated. This highlights the tension between technical and local knowledge, with the latter only being recognized as folklore and not as legitimate knowledge.

*Many people* [referring to professionals] *make comparisons, “Ah, but at the gas station I used to work it was like this, here it’s just dust, it’s dirt”, because there are professionals who go to work in rural areas and don’t want to get dirty with dirt, they don’t want dust* [...]*. And there are many things you have to keep in mind when you go to work in a rural area. Yes, the houses will be far apart, the location will be remote, everything will be far away. The people will be a little closer, there will be many traditions and beliefs that you may not believe in, but you have to think, “So what? Well, that’s fine”, etc. It’s not just in one house that you’ll find someone putting coffee grounds on a wound, it’s every house you go to. You’ll find people with wounds full of coffee grounds because it’s a local belief, so you have to understand that. Don’t judge, just help and show a better way.* (NUR07)

Furthermore, rural life was romanticized, and rural areas were associated with backwardness and a lack of access to technology, even when these communities had socioeconomic conditions similar to urban neighborhoods.

*We need to bring something to them* [the rural population] *to grab their attention, something new* [...] *the issue of technological advancement, right? Many have already felt it. They have access to cell phones, but the issue of types, tablets, research, for instance, some activity that involves technology,* [...] *that’s also important for them to see the reality.* (NUR08)

The use of these technologies can serve as a means of disciplining bodies and knowledge, thereby integrating individuals into the biopolitical apparatus of the state. At the same time, it represents a form of knowledge control mediated by nurses. Nurses have the power to teach and inform, which reaffirms their authority over local ways of life.

Finally, hard lines were also detected in the relationship between nurse and FHS multidisciplinary team.

*Others see me as the leader, the one who coordinates. I try to change that, but I haven’t succeeded yet. They always think things only work if the nurse is in charge. No one ever comes to say, “Look, this month is dedicated to this, let’s do this”. No, it’s always the nurse. A nurse has to say what needs to happen, everywhere, and we’re not recognized for it* [...] *and I don’t think it’s fair that we receive the same pay as other* [professionals] *and work much harder, coordinating the work, but unfortunately, we nurses are a silent cry. It seems like nobody listens to us.* (NUR01)

By describing the lack of recognition and disparity between workload and financial return, nurses reveal the difficult relationship between healthcare professionals and the public system, which operates as a form of subjugation. This subjugation limits professionals’ ability to escape institutional norms that demand rigid adherence to standardized practices and reject creative initiatives adapted to rural contexts.

This statement reveals the effects of a system that assigns multiple responsibilities to rural nurses without proper institutional recognition. It points to a form of subjectivity production marked by individual accountability and the invisibility of collective support. Professionals recognize the limits of their work and express discomfort, highlighting the saturation point of an exhausting model. This perception is understood as a hard line, inscribing the work within a field of burnout.

### Establishment of links as points that produce lines of flight

This discursive chunk illustrates that, despite organizing their work processes with rigid lines, nurses delineate lines of flight that create new paths. Despite the barriers that may arise during the provision of care and cause distancing or other obstacles in serving the rural population, these routes indicate that these events should be seen as opportunities for care. This can be achieved through a reconfiguration of practices to allow for a return to events of division and a new perspective on them, transforming them into opportunities for closer care.

From this perspective, the construction of bonds appears as a point that produces lines of flight, as the following discourse reveals.

*I became too attached to being a rural nurse, also, because I could have a stronger connection with the population* [...] *if there wasn’t that connection, I would only be there as a shift nurse, who goes there, finishes their shift and leaves.* (NUR07)

The powerful recognition of community needs illustrates the weaving of lines of flight that emerge at these breaking points with the established order.

[...] *people have difficulty with transportation, so if there’s a pregnant woman in a remote area, I’ll have to, on the day of the appointment, talk to the driver to arrange to pick her up. I have to coordinate with the driver to pick her up because she has no way to come.* (NUR07)

This bond evades surveillance networks, shifting the emphasis from procedure to relationships and aligning with the humanizing logic of the SUS. The bond becomes powerful when it generates unexpected practices, as illustrated by the inventive care mentioned in the above discourse, since the nurse improvises care networks that cross institutional fields, such as family and transportation. This agency redesigns the territory.

Considering specific practices, the development of bonds is highlighted in prenatal care.

*During prenatal care, we create a bond with the woman, right?* [...] *with the pregnant woman, we have that time with her, the nine months. And then, there are the postpartum check-ups and vaccinations, so there’s that bond with them.* (NUR05)

Even though a prenatal care protocol exists, it has flaws. These flaws include early detection, conversation, and follow-up. These flaws introduce differences within the norm and shift the practice from a purely biomedical approach to a logic of situated care.

Furthermore, the nurses’ role in prenatal care can be seen as that of a cartographer to some extent, since they are interested in and engaged in monitoring the processes inherent to caring for pregnant women.

*I like prenatal care because we plan to follow all the Ministry’s protocols* [...] *the seven appointments, identifying the pregnant woman at the beginning of prenatal care, in the first trimester; performing all the correct exams, all the tests, both the rapid tests and the laboratory exams, the ultrasound exams. When it’s a high-risk pregnancy, we refer her to the high-risk unit* [...]. (NUR01)

The close relationship between those involved in care (caregivers and those receiving care) aligns with the rhizomatic principle of multiplicity in nursing. Nurses must view the population under their care as more than just an object through which they exercise their practice. They must also consider that their practice only exists because of the encounters (lines and networks) that they establish with these individuals in their daily lives. The bonds formed during rural nurses’ care processes function as agencies in which mutual trust allows care to transcend the technical sphere and become more holistic, adapting to the social and cultural particularities of the rural population.

The establishment of strong bonds between rural nurses and the population contributes to their ability to construct a body without organs by weaving lines of flight: a body that, instead of something that must follow certain functions or rules, constitutes a space of potentiality in which intensities and experiences can flow freely. This resists identity hierarchization and promotes a more creative vision of living. It opens up new ways of experiencing and practicing care.

The body without organs provides an opportunity to recreate and reinvent practices within the micropolitics of rural nurses’ work processes based on local needs. This transforms their traditional functions into more open, adaptable, and less controlled practices. By creating a body without organs for themselves, these nurses resist being controlled by social, political, and economic forces that impose limitations. This opens space for inventing new forms of care and subjectivity. This process is a movement of freedom and subversion of power structures, as well as a re-signification of care practices in a rural context.

## DISCUSSION

Providing healthcare to rural populations is challenging due to the geographical dispersion of communities in hard-to-reach areas, different working and living conditions compared to those in urban areas, and the concentration of advanced technologies in large urban centers^(12)^. In light of this, the fundamental role of nurses in promoting the health of rural populations, particularly in remote regions, is evident, underscoring the diverse practices these professionals have developed^(23)^.

The daily work of rural nurses involves uncovering everyday facts and attitudes that require creativity and reflection. This is especially true given the diversity of actions performed by these professionals, who deal with situations that simultaneously facilitate and limit the continuity of care^(12)^.

A contextual analysis of rural nursing care within the scope of PHC revealed that the immediate context of action articulates care activities developed in rural areas. The factors involved in care are addressed within a specific context anchored in a general context based on representations and conceptions that rural nurses and the population jointly develop about care. The shared vision of both regarding knowledge, practice, and care for the promotion of rural health interrelates the previous contexts within a metacontext. This allows for the implementation of targeted, feasible, and potentially effective actions based on knowledge of each contextual layer^(24)^.

The organization of nurses’ work processes and their network of care practices is similar to a care model developed for spontaneous demand in urban FHS settings, as evidenced in another study^(25)^. This study highlights that the frequency of workdays in the area influences the quality of care since professionals have shorter work shifts.

The authors further highlight that these reduced shifts cause care to focus on the biomedical model and medicalization. This approach is ineffective and has a poor social impact due to the absence of actions that prioritize community empowerment regarding healthcare and promote healthy living^(25)^.

From this perspective, the work process is divided, and practice is fragmented into several areas that should form a network of care. Furthermore, the management of care is often confused with the perception that nurses are “responsible for everything”, including nursing procedures and the maintenance of health unit facilities.

Although management is part of care and should be approached with commitment and responsibility in response to the population’s health needs, with the goal of implementing practices that focus on the family and community^(8)^, it is not solely the nurse’s responsibility to manage all dimensions of FHS. This management should be shared with the multidisciplinary team and the administrative sector, in order to avoid perpetuating the image of nurses as a “a jack of all trades”.

This perception is negative for nurses’ professional identity and encourages distancing from the essence of nursing. By being “jacks of all trades”, nurses take on the roles of other professionals and create excessive demands, distancing themselves from actual care^(26)^.

Studies highlight that rural nurses face increasing overload, demands for professional development, and a lack of institutional support^(27)^. In contrast, the adoption of advanced practice nursing models in Organization for Economic Cooperation and Development countries highlights the transformative potential of more autonomous and contextualized practices^(28)^. In Brazil, assessments with Workload Indicators of Staffing Need confirm the prevalence of high workloads and structural gaps that compromise quality of care^(29)^. Although these studies point to similar challenges as those identified in the present study, such as centralization of labor and biomedical models, this study’s cartographic approach highlights the forces capturing care and the lines of flight emerging from bonding and territorialization. This approach offers an analytical lens that is rarely explored in literature on rural PHC and is unprecedented in nursing in this context.

Therefore, we must heed the “silent cry” of rural nurses and recognize the power of their work. We must also join efforts to improve their work processes. Valuing their work means recognizing the power of their care and guaranteeing social prestige, dignified working conditions, salary increases, and institutional respect from society and public administrators. Such appreciation must be anchored in the recognition of care as an essential social practice. As Collière argues^(30)^, caring is a political act that sustains life. However, it has historically been devalued because it is associated with women’s work.

In this same vein, Nightingale^(31)^ proclaimed the importance of care as a pillar of public health and human dignity. She stressed that the effects of nursing cannot be underestimated, nor can its social position be marginalized. Therefore, care must be recognized not only as a technique, but also as an ethical, social, and political practice. Those who perform care work must be valued for all their rights, including material and symbolic ones.

Regarding the hard lines that run through the micropolitics of rural nurses’ work processes, we observed a rigidity of practices centered around spontaneous demand, as well as an absence of shifts focused on scheduled demand and health promotion. This rigidity is partly due to the nursing curriculum, which prioritizes procedures and technical actions as central to continuous learning. This has repercussions that extend beyond training^(32)^.

The rigidity of practices is also discussed by other authors^(33)^, who discuss the importance of flexibility in the schedule for attending to users who seek the health unit, which is carried out from triage or spontaneous demand. This response, however, can lead to fragmentation of care practices, teamwork and, consequently, provide little resolution.

These rigid lines reflect administrative standards and biomedical norms that guide and shape the micropolitics of care through strict schedules and reliance on Ministry of Health protocols. This structure forces nurses to adhere to a limited routine anchored in the biomedical model that is oblivious to the specific needs of the rural population. In this scenario, practices are controlled and standardized, which restricts adaptation and innovation. Rigid lines act as control mechanisms that prevent deviations and configure the healthcare space as a static territory. In this territory, the unique characteristics of rural areas are subordinated to institutional logic.

In this regard, rural nurses miss opportunities to engage in dialogue with the community, understand its needs better, and expand their participation in care planning. The authors^(34)^ point to the need to focus on FHS users as political subjects directly embedded in a context surrounded by needs and possessing knowledge that enables them to contribute to improving services.

Therefore, the importance of the bonds established between the community and rural nurses is reinforced. As researchers point out^(35)^, rural nurses are in a privileged position to build these bonds due to their knowledge of the population and their life stories.

These lines of flight emerge from the connection with the community and operate as ruptures in rigid segmentation. These lines emerge when nurses adapt their practices to strengthen their bond with patients. Home visits, prenatal care, and active listening are moments of deterritorialization. During these moments, care practices break with institutional formality and connect with the real needs of the rural population. It is worth mentioning that rigid segments are defined and socially imposed by the state. In contrast, more flexible segmentation could be viewed as an internal creative or imaginative exercise^(20)^.

In a study of professionals working in FHS in Natal, Rio Grande do Norte, Brazil, the authors highlighted the bond as the main tool for consolidating FHS. They also described it as a fundamental therapeutic resource involving subject-to-subject relationships within existing territorial information networks. The bond is also an essential element for strengthening relationships within the context of family health, establishing itself as a vital care tool^(36)^.

A multicenter study of 40 FHS nurses across five regions of Brazil showed that forming a positive bond with patients facilitates therapeutic action, improves treatment and guidance responses, and helps reduce workloads^(37)^. Other researchers^(25)^ agree, pointing out that the bond is one of the factors that fosters identification with the health context in rural areas and job satisfaction. When activated in a sensitive and situated manner, these bonds create avenues that challenge the established order and open up opportunities for innovation in care.

In the field of professional training and practice, the findings of this study suggest that the rural nurse can be understood as a cartographer of care: someone who follows the living processes of the territory, identifies hard lines that capture work in repetitive routines, and detects lines of flight that allow for the reinvention of practice. This role does not require an understanding of abstract philosophical concepts, but rather, it requires attentive listening, awareness of particularities, and a proactive approach. From this perspective, cartography can be incorporated into nurses’ training as an ethical-political exercise in territorial reading and intervention. This approach encourages situated analyses of the work process and strengthens professional autonomy in the face of institutional regulations.

Based on this analysis, further implications for work management in PHC are highlighted. The rigidity of schedules, the overlapping of functions, and the centralization of planning in nurses-elements recognized as hardline practices-point to the need for management strategies that value active listening to professionals and promote greater organizational flexibility. Enhancing co-management spaces, incorporating participatory processes into agenda-setting, and recognizing the unique characteristics of different territories can foster more creative and thoughtful practices. Therefore, the findings of this study contribute to the rethinking of management models that administer not only resources, but also encourage relationships, autonomy, and creativity in healthcare practices.

### Study limitations

In addition to the limited literature on rural nursing in Brazil, which restricted the discussion of the findings, it should be noted that conducting this study in only one Health Region may limit how applicable the results are to other contexts.

### Contributions to nursing and health

Understanding the lines that run through rural nurses’ work processes provides an opportunity to reflect on ways to improve the health of rural populations and these nurses’ practices. This contributes to shaping rural nursing as a field of knowledge and practice and promotes the advancement of knowledge.

## FINAL CONSIDERATIONS

This study mapped the driving forces underlying the work processes of rural nurses. It revealed rigid schedules, top-down campaigns, and institutional hierarchies that hinder care. However, it also revealed community ties that create opportunities for inventive, holistic practices. These findings offer professional practice insights by suggesting flexible schedules, co-management spaces, and training nurses to map the territory. Furthermore, they contribute to the formulation of public policies by emphasizing the importance of recognizing rural nursing as a strategic field. This recognition should include investment in continuing education, adjustments to workloads, and funding for initiatives that address the unique needs of rural communities, which can significantly contribute to promoting the health and well-being of rural populations.

Furthermore, the use of cartography should be expanded to different regional contexts to explore its analytical and interventional potential. Studies combining qualitative and quantitative methods can assess the effects of possible interventions on community engagement, quality of care, and professional recognition. This strengthens cartography as a management and planning tool in health and contributes to public policies that are more sensitive to local realities.

## Data Availability

The research data are available within the article.
