# Supplementary material for: Cartography of the lines of force that run through rural nurses’ work processes
Source: Rev Bras Enferm. 2026 Jul 17;79:e20250060. doi: 10.1590/0034-7167-2025-0060 (PMC13379193; doi:10.1590/0034-7167-2025-0060)
Supplement: Translation

# Translation: Cartografia das linhas de força que atravessam os processos de trabalho de enfermeiras rurais

**DOI:** 10.1590/0034-7167-2025-0060pt

**Authors:** Bruno Neves da Silva, Nilba Lima de Souza, Deise Lisboa Riquinho, Cristina Andrade Sampaio, Gerlane Cristinne Bertino Veras, Valéria Gomes Fernandes da Silva, Mariana Castilho Valle, Erika Simone Galvão Pinto

**Affiliations:** I Universidade Federal do Rio Grande do Norte. Natal, Rio Grande do Norte, Brasil; II Universidade Federal do Rio Grande do Sul. Porto Alegre, Rio Grande do Sul, Brasil; III Universidade Estadual de Montes Claros. Montes Claros, Minas Gerais, Brasil; IV Universidade Federal de Campina Grande. Cajazeiras, Paraíba, Brasil

**Keywords:** Enfermagem Rural, Atenção Primária à, Saúde, Saúde da População Rural, Cuidados de Enfermagem, Pesquisa Qualitativa.

## Abstract

**Objetivos:**

mapear as linhas de força que atravessam os processos de trabalho de enfermeiras rurais.

**Métodos:**

estudo qualitativo, fundamentado no referencial da cartografia de Deleuze e Guattari. Os dados foram produzidos mediante entrevistas discursivas e da técnica de instrução ao sósia, realizadas entre novembro-dezembro de 2022 e em setembro de 2024, com oito enfermeiras atuantes no sertão paraibano, Brasil. A análise foi orientada pela teorização foucaultiana do discurso.

**Resultados:**

detectaram-se linhas duras limitantes das práticas profissionais, como agendas fixas, campanhas verticalizadas e comparações com cenários urbanos. Em contrapartida, perceberam-se linhas de fuga, manifestas no fortalecimento do vínculo com a comunidade, impulsionando práticas inventivas e adaptações no cuidado.

**Considerações Finais:**

os achados ampliam a ciência da enfermagem ao visibilizar práticas em territórios rurais, oferecendo subsídios analíticos para compreender desafios e singularidades locais. Recomenda-se a formulação de políticas específicas, educação permanente e valorização da enfermagem rural como campo estratégico para o cuidado.

## INTRODUÇÃO

A população rural do Brasil é caracterizada por uma diversidade de etnias, povos, raças, religiões, segmentos econômicos e sociais, além de sistemas de produção, padrões tecnológicos, ecossistemas, e uma vasta e rica biodiversidade. Por conseguinte, a riqueza do Brasil rural está além apenas dos recursos naturais, situando-se também na diversidade do seu povo^(1)^.

Em vista disto, deve ser compreendida mediante as suas inter-relações sociais, político-econômicas e produtivas, e não tão somente como uma população que habita uma área não urbanizada. O território rural, por seu turno, entre outras funções, agrega a agropecuária, a sustentabilidade ecológica e a organização dos grupos sociais, trabalhadores rurais e suas famílias^(2)^.

O cuidado de saúde no meio rural se relaciona a aspectos regionais, demográficos e econômicos que influenciam o planejamento e a gestão da assistência à saúde^(3)^. Quanto à vulnerabilidade da população rural aos problemas de saúde, é percebida, especialmente, pela ausência de cuidados a nível primário, pela falta de saneamento e pelas dificuldades na articulação entre os serviços, o que destaca a relevância de efetivar as políticas públicas no meio rural^(4)^. As políticas públicas de saúde para essas populações, sob tal ponto de vista, precisam se articular de maneira mais integrada com a situação de saúde de cada território^(2)^.

A persistência de desigualdades no processo de cuidado à saúde desenvolvido em âmbito rural aponta para uma dissonância entre o que está sendo realizado e o que se espera da Atenção Primária à Saúde (APS), sendo, muitas vezes, o único recurso de saúde disponível e inserido diretamente no contexto rural brasileiro^(5)^. Tal recurso envolve o desenvolvimento de estratégias voltadas a promover a saúde dos indivíduos, prevenir doenças, e atender às demandas e necessidades reais e potenciais da população rural^(6)^.

A APS no Sistema Único de Saúde (SUS) do Brasil tem sido implementada mediante a Estratégia Saúde da Família (ESF), a qual representou, ainda que com pior desempenho em áreas rurais, um avanço no que se refere à cobertura populacional, mostrando-se uma proposta contra-hegemônica ao modelo biomédico flexneriano predominante no Brasil^(7)^. Nesse cenário, a enfermeira assume papel de destaque como agente articuladora das ações desenvolvidas pela ESF, o que contribui para ampliação dos horizontes de sua atuação profissional^(8)^.

Ainda que não exista consenso quanto à definição de enfermagem rural, autoras a consideram como a prestação de cuidados de saúde, por enfermeiras, a pessoas que vivem em áreas escassamente povoadas^(9)^. A dimensão do fazer em enfermagem rural deve representar uma prática comprometida com a resolução de problemas de cada um dos diversificados contextos, com a construção de diferentes formas de cuidado e com o exercício de refletir criticamente sobre as ações realizadas^(10)^.

No contexto rural, o processo de trabalho da enfermeira demanda um cuidado especializado, pois o cenário possui peculiaridades próprias: territórios geográficos diferenciados e dinâmicos, processos saúde-doença específicos, como a persistência de padrões endêmicos e a baixa qualidade de vida, que exigem a efetivação de medidas para superar os problemas recorrentes na assistência à saúde^(11,12)^. A despeito dessas características, a atuação das enfermeiras rurais é uma maneira efetiva de diminuir as disparidades de saúde^(13-15)^.

Enquanto inseridas na APS e principais responsáveis pela coordenação do cuidado e gerenciamento das Unidades de Saúde da Família, a qualificação das práticas das enfermeiras contribui para o avanço da APS, reforçando que o fortalecimento é a chave para combater as iniquidades em territórios rurais^(5)^. Sob essa ótica, destaca-se a relevância do estudo em tela que, a partir do reconhecimento das linhas de força que atravessam os processos de trabalho de enfermeiras rurais, possibilita refletir sobre a atuação destas e aprimorá-la, a fim de transformar a realidade assistencial, promover a saúde integral da população rural, e corroborar para sua qualidade de vida e bem-viver.

Para alcançar esse objetivo, adotou-se a cartografia como marco conceitual deste estudo, conforme proposta por Deleuze e Guattari. Tal abordagem permite acompanhar os processos vivos da prática profissional e mapear as linhas de força, tensões, repetições e invenções que constituem os cotidianos do trabalho em saúde. Trata-se de abordagem epistêmica ainda pouco explorada no cenário nacional e ausente no debate nacional e internacional sobre a enfermagem em contextos rurais. Sem a pretensão de esgotar o tema, o estudo inaugura um possível caminho para constituição de outras formas de compreender a prática profissional das enfermeiras rurais sensíveis às singularidades do cuidado e às forças que o atravessam. Diante do exposto, buscou-se responder à questão teórica: como as linhas de força atravessam a organização dos processos de trabalho de enfermeiras rurais?

## OBJETIVOS

Mapear as linhas de força que atravessam os processos de trabalho de enfermeiras rurais.

## MÉTODOS

### Aspectos éticos

Este estudo deriva da tese de doutorado intitulada “Micropolítica dos processos de trabalho de enfermeiras rurais: uma cartografia das práticas de cuidado”. Foi aprovado pelo Comitê de Ética em Pesquisa da Universidade Federal do Rio Grande do Norte, sob Parecer n^o^ 5.695.462. A pesquisa foi balizada pelas Resoluções n^o^ 466/2012 e n^o^ 510/2016 do Conselho Nacional de Saúde do Brasil. A anuência das participantes do estudo ocorreu mediante explicação dos objetivos, leitura e assinatura do Termo de Consentimento Livre e Esclarecido em duas vias. O anonimato das interlocutoras da pesquisa foi garantido, e, para utilização de suas falas no decorrer do texto, foi utilizado o codinome “ENFR”, seguido de um número cardinal, conforme a ordem de realização de cada procedimento de produção de dados.

### Referencial teórico-metodológico

Este estudo se ancora na cartografia de Gilles Deleuze e Felix Guattari. A cartografia está vinculada ao pensamento pós-estruturalista, e representa uma abordagem metodológica contemporânea que pode ser interpretada como metodologia, método ou procedimento metodológico, a depender da aplicação, das intenções do pesquisador e do papel que desempenha no processo de pesquisa^(16)^.

O termo “cartografia”, em Deleuze e Guattari, sugere que as malhas que compõem um rizoma não possuem profundezas a serem perscrutadas, mas se engendram em linhas que se emaranham na construção de tramas, compondo diversificadas trajetórias forjadas em distintas intensidades (políticas, linguísticas, estéticas, econômicas, biológicas, entre outras), as quais indicam movimentos que constituem um mapa de intensidades e afetos no formar de territórios existenciais^(17)^.

Autores^(18)^ destacam que o estudo dessas linhas é importante para compreensão dos processos de subjetivação. Tratam-se das relações de forças de sentidos que são tecidas pelos sujeitos em sociedade e no mundo, sendo linhas que escapam, frequentemente, aos próprios sujeitos, delineiam outras experiências de sentido ou tornam caóticas as que já existem.

As linhas de segmentaridade dura são características dos grandes estratos ou conjuntos molares (classes sociais, gêneros, percurso família-escola, escola-trabalho, etc.). São assinaladas pela rigidez e relacionadas à própria formação do sujeito. São linhas que esboçam classificações (como classe, nível, etc.) e funcionam de modo dicotômico, aludindo ao aspecto da realidade que se mostra como dado, naturalizado, instituído, e que apresentam caráter permanente, cuja tendência é afastar a crítica e o questionamento^(18,19)^.

As linhas de fuga, no que lhes concerne, constituem linhas de ruptura que desfazem o “eu” com suas relações estabelecidas e o conduzem à pura experimentação do devir, ainda que de forma momentânea. Frequentemente, as linhas de fuga precisam ser inventadas, sem um modelo orientador, e representam as linhas que liberam o desejo do aprisionamento dos estratos, necessitando, por conseguinte, ser produzidas nas cartografias dos sujeitos, sobretudo quando seu potencial se depara ameaçado por estratificações acentuadas^(18)^.

Essas linhas possibilitam escapes e resistências ao instituído, definindo as multiplicidades e assinalando a realidade de um número finito de dimensões que são, de fato, preenchidas por esta^(20)^.

### Tipo de estudo

Estudo qualitativo. Utilizou-se o *Standards for Reporting Qualitative Research* como guia para orientar o relatório de pesquisa, conforme requisito do periódico.

### Procedimentos metodológicos

Foram incluídas no estudo enfermeiras com atuação igual ou superior a um ano em territórios rurais, tempo julgado necessário para que as profissionais elaborassem sua identidade como enfermeiras rurais. Foram excluídas do estudo profissionais que, durante o período de produção dos dados, estivessem ausentes dos serviços por motivos de licença, férias, afastamento, entre outros. A partir da observância dos critérios de seleção, o contato com as participantes se deu face a face, a partir de amostragem teórica.

### Cenário do estudo

O estufo foi realizado em unidades rurais de ESF localizadas na 10ª Regional de Saúde paraibana, Brasil.

### Coleta e organização dos dados

Os dados foram produzidos com as interlocutoras selecionadas no período de novembro a dezembro de 2022 e setembro de 2024, sendo o processo realizado por um pesquisador enfermeiro com grau de mestrado, com leitura no referencial adotado e experiência em estudos com abordagens qualitativas. O processo ocorreu nas salas em que atuavam as enfermeiras nas unidades de ESF, local indicado por elas, por ser de fácil acesso e representar um ambiente considerado adequado e reservado para o momento.

A produção de dados ocorreu por intermédio de entrevistas discursivas. Utilizou-se um roteiro com perguntas abertas como guia do processo, o qual não atuou como um instrumento normatizador, mas como um operador performativo, capaz de ativar sentidos em fluxo na tessitura da entrevista. De forma complementar às entrevistas, recorreu-se à técnica de instrução ao sósia, que consistiu em dar o seguinte comando para as interlocutoras: “Imagine que eu sou seu sósia e amanhã irei substituí-la no trabalho. Quais instruções você me daria para que ninguém notasse a substituição?”.

Trata-se de técnica oriunda da psicologia e coerente com as intenções do estudo em tela, especialmente adequada à cartografia, por favorecer o exercício de uma fala encarnada, situada e reflexiva, que potencializa a emergência de afetos, gestos e saberes que atravessam as práticas. Mediante a utilização dessa técnica, ampliou-se o alcance analítico, produzindo-se um deslocamento na narrativa, o que propiciou acesso às singularidades do trabalho para além dos enunciados convencionais, em consonância com o interesse cartográfico de acompanhar os modos de existir em sua processualidade.

O critério de interrupção da produção dos dados foi guiado pela recorrência de certos efeitos de sentido e pela percepção de forças que constituíram um campo analítico coerente com o objetivo do estudo e com os princípios cartográficos. No campo da cartografia, não se busca a saturação dos dados, uma vez que a pesquisa não parte da representação ou da totalidade, mas sim do acompanhamento dos processos em sua multiplicidade e movimento. Além disso, à luz dos preceitos da análise do discurso, a possibilidade de alcançar uma saturação plena é questionável, uma vez que uma dada ordem discursiva, situada em seu tempo, permanece continuamente suscetível a (re)interpretações *a posteriori*.

O tempo mínimo de duração das entrevistas foi de 12 minutos, e o máximo foi de 34 minutos. Foram realizadas oito entrevistas, que foram audiogravadas e posteriormente transcritas, dando origem ao *corpus* de pesquisa.

### Análise dos dados

A produção e análise de dados foram simultâneas, recorrendo-se às teorizações acerca do discurso em Michel Foucault para análise. Segundo o autor, a análise do discurso não desvenda o sentido universal das coisas ditas, mas apresenta, à luz do dia, a trama de rarefações impostas, com um poder fundamental de afirmação^(21)^. Sob a ótica foucaultiana, analisar o campo discursivo consiste em compreender o enunciado na sua singularidade e especificidade situacional, identificar as condições de sua existência, delimitar adequadamente seus contornos, estabelecer suas conexões com outros enunciados aos quais pode estar associado, e demonstrar quais formas de enunciação são excluídas. Não se procura, por trás do que está explícito, a conversa silenciosa de um discurso diferente. É necessário mostrar por que não poderia ser de outra forma, como exclui qualquer possibilidade alternativa, como ocupa, entre as outras e em relação a elas, um lugar único que nenhuma outra poderia ocupar^(22)^.

Nessa perspectiva, a análise operou por meio da identificação de regularidades discursivas nos enunciados das participantes, bem como dos efeitos de verdade que sustentam determinadas formas de cuidar e de ser enfermeira rural. Procedeu-se à construção de blocos discursivos, nos quais foram agrupados fragmentos que expressavam linhas de força, como normas, rupturas e resistências nos processos de trabalho. Essa operação analítica se articulou ao referencial da cartografia, permitindo acompanhar o aparecimento de discursos no plano micropolítico e mapear suas implicações nas práticas profissionais.

A construção desses blocos não seguiu etapas fixas ou categorização prévia. Os blocos foram elaborados no movimento de análise imanente aos dados, em consonância com a cartografia, o que implicou o acompanhamento dos efeitos de sentido que se atualizavam nos encontros entre o pesquisador, os discursos e o campo, atentos às intensidades, repetições e variações. Os blocos foram organizados reconhecendo-se linhas de força que abrangiam os processos de trabalho, às vezes como repetições normativas (linhas duras) e outras vezes como variações inventivas (linhas de fuga), o que permitiu visualizar zonas de tensão, deslocamento ou reinvenção nas práticas analisadas.

## RESULTADOS

Após uma recusa por falta de tempo, participaram do estudo sete enfermeiras e um enfermeiro rural, com idade mínima de 28 e máxima de 56 anos. Sete possuíam especialização em diversas áreas da enfermagem, incluindo APS, e uma possuía mestrado. O tempo de atuação na zona rural foi de um a 14 anos. Todas as interlocutoras declararam não ter participado de nenhuma formação específica para atuar na zona rural antes ou depois da inserção na prática. Optou-se pelo uso do gênero feminino no plural, em consonância com a composição majoritariamente feminina do estudo e com a prevalência histórica e sociocultural de mulheres na enfermagem.

Quanto aos dados provenientes das entrevistas e da técnica de instruções ao sósia, a cartografia compôs dois blocos discursivos, sendo cada enunciado analisado de acordo com sua condição de possibilidade no regime de saber-poder e com sua inscrição em linhas duras ou linhas de fuga.

### “Tem profissional da zona rural que não quer se sujar de terra”: as linhas duras que atravessam a micropolítica dos processos de trabalho da enfermeira rural

Compreendeu-se que os enunciados que circundavam os discursos das profissionais materializavam linhas de segmentaridade duras, engessando as práticas de cuidado. Exemplo disso são agendas ou cronogramas mensais fixos, sem demanda programada, e a não menção à consulta de enfermagem como parte do processo de trabalho assistir em enfermagem. O contexto analisado revelou que o planejamento semanal funciona como tecnologia disciplinar que fragmenta o tempo, classifica corpos e legitima um olhar médico-centrado. O mesmo existe apenas por ser sustentado por dispositivos ministeriais, protocolos e indicadores que definem o que conta como cuidado “cientificamente correto”, dificultando devires inventivos capazes de responder às singularidades do território. O discurso a seguir ilustra esse atravessamento na micropolítica.

*Minha carga horária é de 40 horas semanais, de segunda à sexta. Às segundas* [manhã]*, é feito o atendimento pré-natal, à tarde, demanda espontânea. Na terça de manhã, é relacionado a testes rápidos, e, de tarde, demanda espontânea. Na quarta de manhã, temos a puericultura, e, à tarde, demanda espontânea. Na quinta-feira, a gente tem os atendimentos de vacinação, e, à tarde, visita domiciliar. E na sexta, a gente realiza o HIPERDIA, que é o atendimento aos hipertensos e diabéticos. Na sexta à tarde, eu tenho uma folga.* [...] *na demanda espontânea, aparece questão de curativos, administrar alguma medicação* [...]. (ENFR02)

O tramar dessas linhas é reforçado por um modelo campanhista ditado pela gestão, que privilegia ações como “Outubro Rosa” ou “Novembro Azul”, distanciando-se de demandas específicas da população rural, como a promoção da saúde dos trabalhadores do campo. Embora beneficiem a população, tais campanhas funcionam como rituais de verdade e delimitam quem deve ser educado, de que forma, sobre quais temas, quais exames importam e quando isso deve acontecer, o que reforça a autoridade institucional das enfermeiras rurais.

Temas como agrotóxicos, saúde do trabalhador ou violências rurais permanecem fora do campo de enunciação, devido a não se encaixarem nos códigos de vigilância que sustentam o financiamento e as metas da unidade. Cada campanha é configurada como linha dura, tecendo uma malha que captura fluxos de cuidado em datas fixas, repete discursos e restringe devires inventivos que dialoguem com as especificidades do território. Essas ações esbarram em horários pouco acessíveis, temas pré definidos e na lógica numérica de atendimentos, o que dificulta a escuta das singularidades rurais.

[...] *é mais tipo campanha* [as práticas educativas]. *É aqui na sala de espera, “Outubro Rosa”, “Novembro Azul”. O nosso “Novembro Azul” vai ser agora, na última quinta-feira do mês* [...] *vamos fazer a palestra educativa com o médico, e os usuários que vierem terão o exame PSA solicitado, e eu ficarei fazendo os testes rápidos também.* (ENFR01)

Ainda que a gestão dite os moldes das campanhas educativas, há uma reatualização desses modelos pela atitude passiva das enfermeiras, que são responsáveis pela construção dos seus cronogramas mensais. Como observado nos discursos anteriores, esses cronogramas geralmente não incluem práticas educativas no cotidiano, além das ações vinculadas às campanhas. Ademais, observa-se um discurso que culpabiliza os usuários pela baixa adesão às ações de promoção da saúde, isentando a profissional de sua parcela de responsabilidade.

[...] *a gente, para fazer campanha, precisa ter que estar ofertando alguma coisa diferente para as pessoas virem, mas elas não querem perder tempo.* (ENFR01)

[...] *a gente marca* [ações educativas], *mas o povo aqui não é muito de palestra* [...] *não dão valor a isso não.* (ENFR05)

Contudo, há discursos que contradizem a ideia de que a população não valoriza ações educativas. Em alguns casos, a população demonstrou grande adesão às atividades quando estas se alinharam às suas possibilidades.

*Chegamos a fazer uma ação do “Outubro Rosa”, a primeira ação noturna, e foi, assim* [...] [gesticula com as mãos]*: a gente ficou surpresa! Até a secretária* [de saúde] *ficou surpresa com o volume do público!* (ENFR08)

A população aparece como sujeito carente de educação, e a recusa de participação da comunidade que aparece nos discursos anteriores confirma a autoridade profissional. O contraponto da comunidade referido no discurso acima rompe essa verdade, denunciando que a ausência era efeito dos formatos impostos, não de desinteresse.

Ao se referir à população, a enfermeira rural fala a partir de uma posição de autoridade no campo da saúde, uma vez que é uma profissional treinada e, portanto, detém um saber legitimado e reconhecido socialmente. Esse saber atribui um lugar de poder na relação que circula pelo discurso. A população rural é descrita de forma homogênea e em termos de uma falta (“não dão valor”), o que reforça uma relação hierárquica implícita, sendo posicionada como carente de saber ou de interesse no que a enfermeira considera importante. Destarte, ao invés de dialogar com os saberes e práticas contextuais, a prática discursiva das enfermeiras denota que se espera que a população rural adote a lógica do modelo biomédico sem uma verdadeira adaptação contextual.

Ademais, a perda de oportunidades de diálogo para estabelecimento de vínculos com a comunidade é revelada nos discursos e evidenciada nos cronogramas que incluem turnos inteiros de “demanda livre” pouco aproveitados para ações participativas. A própria ausência de reuniões comunitárias revela uma lacuna. Destarte, a enfermeira reconhece a ausência de participação social, prática historicamente subalternizada no dispositivo biomédico, o que sinaliza uma ruptura enunciativa, na qual se percebe que o cuidado vem sendo produzido sem a voz da comunidade. A possibilidade de reunião explicita que o enunciado dominante depende da invisibilidade do outro.

[...] *aqui, para a comunidade, nós nunca marcamos uma reunião* [...] *é até uma boa, né? Chamar a comunidade para participar, mas as pessoas não querem perder tempo, não* [...] *infelizmente, a maior dificuldade é essa.* (ENFR01)

Percebeu-se, ainda, uma perturbação ocasionada pelo pesquisador-cartógrafo na prática discursiva da enfermeira no trecho “é até uma boa, né?”, que funcionou como uma microintervenção nos modos de fazer da profissional, deslocando a do automatismo da agenda. Tal perturbação confirma a potência da cartografia não apenas como método de observação, mas como dispositivo que, ao tocar o real, produz movimentos ético políticos no interior do processo de trabalho “assistir em enfermagem”.

Linhas duras também foram detectadas no fluxo de atendimento, que tende a esvaziar a autonomia da enfermeira. Em geral, os usuários são triados por técnicas de enfermagem, que decidem se há necessidade de encaminhamento para enfermeiras, médicas ou dentistas. Essa triagem define uma hierarquia entre saberes e lugares institucionais. Essa organização reforça a centralidade do saber médico, deslocando a consulta de enfermagem para um plano acessório, o que configura uma redistribuição funcional baseada em uma epistemologia médico-biológica.

*O fluxo geralmente é esse: o paciente é atendido pela recepção, e aí, a partir daí, ele vai ser encaminhado para a triagem; passa pela avaliação da técnica de enfermagem, e lá vai fazer essa triagem, avaliando também qual a necessidade de atendimento, se é ou não urgente* [...] *aí vai ser encaminhado para a enfermeira, para o médico, ou para o dentista.* (ENFR02)

Apesar da crítica à biomedicalização presente implicitamente nos seus discursos, as enfermeiras rurais aderem aos protocolos e os reproduzem, visto que estes se tornam uma condição *sine qua non* para um atendimento considerado adequado. Nesse contexto, não são apenas instrumentos técnicos, mas artefatos de poder de caráter normativo que conferem legitimidade à prática profissional. Questioná-los implica riscos simbólicos e institucionais, como a desqualificação da enfermeira ou a aplicação de sanções simbólicas ou práticas no interior da equipe ou gestão. Destarte, mesmo diante do incômodo com os limites impostos pelos protocolos biomédicos, as enfermeiras frequentemente os reproduzem, pois neles se ancora a possibilidade de reconhecimento técnico, pertencimento institucional e segurança legal de sua atuação.

Dessa forma, o campo da enfermagem rural se torna atravessado por uma tensão constante. De um lado, há potência do cuidado situado, afetivo e inventivo, mas, de outro, a necessidade de conformidade com uma lógica normatizadora que padroniza os corpos, os saberes e os gestos. Nessa ambivalência, perpetua-se a manutenção do *status quo*, em que práticas criativas ou desviantes são inibidas, e o cuidado se vê cada vez mais capturado por indicadores, metas e formatos predefinidos que nem sempre respondem às singularidades da vida rural.

No percurso analítico, linhas duras também foram detectadas a partir das concepções pessoais das profissionais enfermeiras e comparações entre os processos de trabalho na zona rural e na zona urbana. O enunciado delimita o dentro/fora do discurso sanitário, em que poeira, crença e tradições são aspectos tidos como irracionais, porém tolerados, o que destaca uma tensão entre o saber técnico e o saber local, que só é audível enquanto folclore, não como conhecimento legítimo.

*Muita gente* [referindo-se aos profissionais] *fica fazendo comparativos: “Ah, mas no posto que eu trabalhava era assim, aqui é só poeira, é terra”* [...] *porque tem profissional que vai trabalhar em zona rural e não quer se sujar de terra, não quer que tenha poeira* [...]. *E aí, tem muita coisa que você tem que levar na mente quando você vai trabalhar na zona rural. Sim, as casas vão ser distantes, o local vai ser distante, tudo vai ser longe. O pessoal vai ser um pouco mais vizinho, vão ter muitas tradições e crenças que você pode não acreditar, mas você tem que “E é? Pois está bom” e etc. Não é só em uma casa que você vai achar alguém que vai botar pó de café na ferida, é toda casa que você chegar. Vai achar o povo com ferida cheia de pó de café, porque é uma crença local, então você vai ter que entender isso. Não julgar e só auxiliar e mostrar uma forma melhor.* (ENFR07)

Além disso, foi observada uma romantização da ruralidade, que associa o rural ao atraso e à falta de acesso à tecnologia, mesmo quando essas comunidades têm condições socioeconômicas semelhantes às de bairros urbanos.

*Tem que levar para eles* [população rural] *algo para chamar mais a atenção, de deixa eu ver, algo novo* [...] *a questão do avanço tecnológico, né? Muitos já sentiram. Eles têm acesso a celular, mas a questão de tipo, de tablet, de pesquisa, de... um exemplo, alguma atividade que envolva a parte tecnológica,* [...] *isso é importante, também, para eles verem a realidade.* (ENFR08)

Esse uso das tecnologias pode funcionar como ferramenta de disciplinarização dos corpos e dos saberes, integrando os sujeitos ao aparato biopolítico do Estado. Ao mesmo tempo, representa uma forma de controle sobre o saber, mediado por enfermeiras, que detêm o poder de ensinar e informar, reafirmando sua autoridade sobre os modos de vida locais.

Por fim, linhas duras também foram detectadas na relação entre a enfermeira e a equipe multidisciplinar da ESF.

*Os outros me enxergam como a líder, a que coordena. Eu tento tirar isso, mas ainda não consegui. Sempre pensam que as coisas só funcionam se a enfermeira estiver à frente. Ninguém nunca chegou para dizer: “Olhe, esse mês é destinado a isso, vamos realizar isso”. Não, é sempre a enfermeira. A enfermeira que tem que dizer que tem que acontecer, em todo lugar, e a gente não é reconhecido por isso* [...] *e eu não acho justo a gente receber igual aos demais* [profissionais] *e trabalhar bem mais, a articulação dos trabalhos, mas aí, infelizmente, nós enfermeiras somos um grito de silêncio. Parece que ninguém escuta a gente não.* (ENFR01)

Ao descrever a falta de reconhecimento e a disparidade entre a carga de trabalho e o retorno financeiro, a enfermeira mapeia uma linha dura na relação dos profissionais de saúde com o sistema público, que opera como uma forma de sujeição. Essa sujeição limita a capacidade das profissionais de escaparem das normas institucionais, exigindo um cumprimento rígido das práticas padronizadas, bloqueando iniciativas mais criativas ou adaptadas ao contexto rural.

O sentimento de sobrecarga expresso nesse enunciado desvela ainda os efeitos de um dispositivo que atribui às enfermeiras rurais múltiplas responsabilidades, sem o devido reconhecimento institucional, o que aponta para uma forma de produção de subjetividade marcada pela responsabilização individual e pela naturalização da invisibilidade do apoio coletivo. A profissional reconhece os limites de sua atuação, expressando um incômodo que evidencia o ponto de saturação de um modelo exaustivo. Essa percepção é compreendida como uma linha dura, que inscreve o trabalho em um campo de esgotamento.

### O estabelecimento de vínculos como pontos produtores de linhas de fuga

Este bloco discursivo retrata que as enfermeiras, apesar de organizarem os seus processos de trabalho em um emaranhado de linhas duras, delimitam pontos de escape que tramam novos percursos. Esses pontos indicam que, apesar das barreiras que possam surgir durante a prestação de cuidados, e que possam causar afastamento ou outros obstáculos no atendimento à população rural, elas devem ser vistas como eventos capazes de gerar novas oportunidades de cuidado, por meio de uma reconfiguração de práticas, a fim de permitir, entre outras coisas, um retorno aos eventos de divisão e vê-los sob outra perspectiva, transformando-os em oportunidades para um cuidado mais próximo.

Nessa perspectiva, a construção de vínculos aparece como ponto produtor de linhas de fuga, conforme desvela o discurso a seguir.

*Eu me apeguei demais a ser uma enfermeira rural, também, por questão de eu conseguir ter mais vínculo com a população* [...] *se não houvesse o vínculo, eu estaria lá só como enfermeira de plantão, que vai lá, passa o plantão e vai embora.* (ENFR07)

O tramar das linhas de fuga que surgem nesses pontos de ruptura com as linhas duras é ilustrado pelo reconhecimento potente das necessidades comunitárias.

[...] *as pessoas têm dificuldade de transporte, então, se tiver uma gestante em um sítio distante, eu vou ter que, no dia do atendimento, eu vou falar com o motorista para poder ir buscar. Eu tenho que articular com o motorista para ir buscar ela, porque ela não tem como vir.* (ENFR07)

O vínculo escapa às malhas de vigilância e desloca a ênfase do procedimento para a relação, inscrevendo-se na lógica humanizadora do SUS. Torna se potente quando gera práticas inesperadas, como ilustrado no cuidado inventivo aludido no discurso acima, visto que a enfermeira improvisa redes de cuidado que atravessam campos institucionais, como família e transporte, com um agenciamento que redesenha o território.

Considerando-se as práticas específicas, a produção de vínculos é destacada no acompanhamento pré-natal.

*No pré-natal, a gente cria um vínculo com a mulher, né?* [...] *a gestante, a gente tem aquele tempo com ela, os nove meses. E depois, ainda vêm a consulta puerperal e as vacinas, então tem aquele vínculo com elas.* (ENFR05)

Ressalta-se que, ainda que exista um protocolo de acompanhamento pré-natal, mesmo neste existem fissuras, como captar cedo, conversar e acompanhar, gestos que introduzem diferenças no interior da norma, originando linhas de fuga que deslocam a prática do eixo biomédico puro para uma lógica de cuidado situado.

Além disso, no acompanhamento pré-natal, em específico, visualiza-se, em algum grau, a posição da enfermeira como cartógrafa, uma vez que estas se interessam e se engajam no acompanhamento dos processos inerentes ao cuidado à gestante:

*Eu gosto do pré-natal porque a gente planeja para seguir todo o protocolo do Ministério* [...] *as sete consultas, captar a gestante no início do pré-natal, no primeiro trimestre; realizar todos os exames certinhos, todos os exames, tanto as testagens, os testes rápidos e os exames laboratoriais, os exames de ultrassom. Quando é gestante de alto risco, encaminhar para o alto risco* [...]. (ENFR01)

Essa aproximação que ocorre entre os sujeitos implicados no cuidado (o ser cuidador e o ser sob cuidados) aproxima o desenvolvimento pela enfermeira do princípio rizomático da multiplicidade. A enfermeira deve encarar sua população sob cuidados não apenas como um fim em si mesma ou um objeto através do qual ela exerce sua prática, mas considerar que essa prática só existe devido aos encontros (linhas e tramas) que esses sujeitos estabelecem consigo no seu cotidiano. Os vínculos nos processos de cuidado das enfermeiras rurais funcionam como agenciamentos nos quais a confiança mútua permite que o cuidado ultrapasse a esfera técnica e se torne mais integral, adaptando-se às particularidades sociais e culturais da população rural.

A constituição de vínculos potentes entre as enfermeiras rurais e a população, no tramar das linhas de fuga, contribui ainda para que possam construir para si um corpo sem órgãos: um corpo que, em vez de algo que deve seguir certas funções ou regras, constitui-se em um espaço de potencialidade em que as intensidades e experiências podem fluir de forma livre, resistindo à hierarquização identitária e promovendo uma visão mais criativa do viver, abrindo-se para novas formas de experienciar e praticar o cuidado.

O corpo sem órgãos oferece a possibilidade de recriar e reinventar as práticas na micropolítica dos processos de trabalho das enfermeiras rurais, a partir das necessidades locais, transformando suas funções tradicionais em práticas mais abertas, adaptáveis e menos controladas. Ao criar para si um corpo sem órgãos, estas resistem à captura por forças sociais, políticas e econômicas que promovem limitações, abrindo espaço para invenção de novas formas de cuidado e subjetividade. Esse processo constitui-se em um movimento de liberdade, de subversão às estruturas de poder, e de ressignificação das práticas de cuidado no contexto rural.

## DISCUSSÃO

O cuidado de saúde às populações rurais configura uma demanda complexa, devido à dispersão territorial das comunidades em áreas de difícil acesso, às condições de trabalho e de vida distintas das condições urbanas, à concentração de tecnologias mais avançadas nos grandes centros urbanos, entre outros fatores^(12)^. À vista disso, destaca-se o papel fundamental da enfermeira na promoção da saúde da população rural, especialmente nas regiões de difícil acesso, evidenciando-se o amplo escopo de práticas desenvolvidas por essas profissionais^(23)^.

O prisma do cotidiano laboral das enfermeiras rurais implica o desvelamento de fatos e atitudes do dia a dia que demandam criação e reflexão, sobretudo pela diversidade de ações desenvolvidas, uma vez que essas profissionais lidam com situações que, simultaneamente, facilitam e limitam a continuidade do cuidado^(12)^.

Análise contextual do cuidado da enfermeira rural no âmbito da APS revelou que o seu contexto imediato de ações articula as atividades de cuidado desenvolvidas em território rural, e os fatores intervenientes no cuidado são interpelados em um contexto específico, ancorado em um contexto geral que se alicerça nas representações e concepções que a enfermeira rural e a população elaboram conjuntamente sobre o cuidado. A visão compartilhada entre ambos sobre o saber/fazer/cuidar para promoção da saúde rural inter-relaciona os contextos anteriores em um metacontexto, o que permite, a partir do conhecimento de cada camada contextual, a realização de ações direcionadas, mais exequíveis e possivelmente resolutivas^(24)^.

A organização do processo de trabalho da enfermeira e sua malha de práticas cuidativas se aproxima de um modelo de atendimento à demanda espontânea desenvolvido nos cenários de ESF urbana, conforme já evidenciado em outro estudo^(25)^, que destaca que a periodicidade de dias de trabalho na área acaba por influenciar a qualidade assistencial, uma vez que as profissionais possuem turnos de trabalhos reduzidos.

Os autores destacam ainda que, uma vez que esses turnos se encontrem diminuídos, a assistência se focaliza no modelo biomédico e na medicalização, com resolutividade baixa e precário reflexo social, devido à ausência de ações que privilegiem o empoderamento da comunidade no que diz respeito aos cuidados de saúde com vistas à promoção de um viver saudável^(25)^.

Nessa perspectiva, o processo de trabalho é marcado por cisões, e a prática se encontra fragmentada em diversos pontos que deveriam constituir um rizoma de cuidados. Ademais, o processo de gerência do cuidado tem sido confundido com a percepção de que a enfermeira é “responsável por tudo”, desde os procedimentos de enfermagem até a manutenção da estrutura física das unidades de saúde.

Embora a gestão faça parte do cuidado e deva ser exercida com compromisso e responsabilidade diante das necessidades de saúde da população, visando à efetivação de práticas que tenham a família e o coletivo como focos centrais^(8)^, não é responsabilidade exclusiva da enfermeira gerir todas as dimensões da ESF. Essa gestão deve ser compartilhada com a equipe multiprofissional e com o setor administrativo, a fim de evitar a perpetuação da imagem da enfermeira como uma “faz-tudo”.

Tal percepção, além de negativa para a sua identidade profissional, incentiva o distanciamento com a essência da enfermagem, uma vez que, ao “fazer tudo”, a enfermeira assume papeis de outros profissionais, além de proporcionar demanda excessiva e distanciamento do cuidado propriamente dito^(26)^.

Estudos destacam que as enfermeiras rurais enfrentam crescente sobrecarga, exigência por desenvolvimento profissional e escassez de apoio institucional^(27)^. Em contrapartida, a adoção de modelos de Enfermagem de Prática Avançada em países da Organização para a Cooperação e Desenvolvimento Econômico evidencia o potencial transformador de práticas mais autônomas e contextualizadas^(28)^. No Brasil, avaliações com indicadores *Workload Indicators of Staffing Need* (WISN) confirmam a prevalência de cargas elevadas e lacunas estruturais que comprometem a qualidade do cuidado^(29)^. Embora esses estudos apontem para desafios semelhantes aos identificados no estudo em tela, como centralização do trabalho e modelos biomédicos, a abordagem cartográfica adotada no presente estudo destaca as forças que capturam o cuidado e as linhas de fuga que emergem a partir do vínculo e da territorialização, oferecendo uma lente analítica ainda pouco explorada na literatura sobre a APS rural e inédita no campo da enfermagem nesse contexto.

Urge, em vista disso, ouvir o “grito de silêncio” das enfermeiras rurais, em busca de reconhecer a potência da sua atuação e somar esforços para qualificar os seus processos de trabalho. Valorizar a sua atuação significa não apenas reconhecer a potência do cuidado que produzem, mas garantir prestígio social, condições dignas de trabalho, valorização salarial e respeito institucional por parte da sociedade e dos gestores públicos. Tal valorização deve estar ancorada no reconhecimento do cuidado como prática social essencial, como defende Collière^(30)^, ao afirmar que cuidar é um ato político que sustenta a vida, mas que historicamente tem sido desvalorizado por estar ligado ao trabalho feminino.

Nightingale^(31)^, nessa mesma direção, proclamou a importância do cuidado como pilar da saúde pública e da dignidade humana, e ressaltou que a enfermagem não pode ser subestimada em seus efeitos, nem marginalizada em sua posição social. O cuidado, portanto, precisa ser reconhecido não apenas como técnica, mas como prática ética, social e política. Destarte, quem o realiza precisa ser valorizada em todos os seus direitos, inclusive materiais e simbólicos.

Quanto às linhas duras que atravessam a micropolítica dos processos de trabalho da enfermeira rural, observaram-se um engessamento das práticas ao redor da demanda espontânea e a ausência de turnos voltados à demanda programada e promoção da saúde. Parte desse engessamento se dá em virtude do currículo de enfermagem, que prioriza procedimentos e ações técnicas como fatores centrais da aprendizagem contínua, o que ocasiona reflexos para além da formação^(32)^.

O engessamento das práticas também é discutido por outras autoras^(33)^, que discorrem acerca da importância da flexibilização do cronograma para atendimento de usuários que buscam a unidade de saúde, o que é realizado a partir do atendimento de triagem ou de demanda espontânea. Essa resposta, no entanto, pode ocasionar fragmentação das práticas de cuidado, do trabalho em equipe e, consequentemente, proporcionar pouca resolutividade.

As linhas duras refletem os padrões administrativos e as normativas biomédicas que guiam e moldam a micropolítica do cuidado, mediante cronogramas rígidos e a dependência dos protocolos do Ministério da Saúde. Essa estrutura obriga enfermeiras a seguir uma rotina limitada, ancorada no modelo biomédico e alheia às especificidades da população rural. Nesse cenário, as práticas são controladas e normalizadas, restringindo a adaptação e a inovação. As linhas duras atuam, assim, como mecanismos de captura que impedem desvios e configuram o espaço da saúde como um território estático, no qual as singularidades do contexto rural são subordinadas à lógica institucional.

Nesse sentido, as enfermeiras rurais perdem a oportunidade de estabelecer maior diálogo com a comunidade, entender melhor as necessidades e ampliar sua participação no planejamento do cuidado. Autoras^(34)^ sinalizam para a necessidade de enfocar os usuários da ESF não como objetos de ação, mas como sujeitos políticos diretamente inseridos em um contexto, rodeados de necessidades e detentores de saberes que lhe possibilitam contribuir para a melhoria dos serviços.

Destarte, reforça-se a importância dos vínculos estabelecidos entre a comunidade e a enfermeira rural, profissional que, conforme destacam pesquisadores^(35)^, possui localização privilegiada para construí-los, devido ao seu conhecimento da população e às suas histórias de vida.

As linhas de fuga, inclusive, são percebidas a partir desse processo de vinculação com a comunidade, operando como rupturas na segmentaridade dura. Essas linhas surgem quando as enfermeiras adaptam práticas para fortalecer o vínculo com os usuários. As visitas domiciliares, o acompanhamento pré-natal e as práticas de escuta qualificada representam momentos de desterritorialização, em que as práticas de cuidado rompem com a formalidade institucional e se conectam às necessidades reais da população rural. É válido mencionar que os segmentos rígidos são definidos e socialmente impostos, sendo sobrecodificados pelo Estado. Em contrapartida, a segmentação mais flexível poderia ser vista como um exercício interno, criativo ou imaginativo^(20)^.

Em estudo com profissionais atuantes na ESF de Natal, Rio Grande do Norte, Brasil, autores evidenciaram o vínculo como principal ferramenta de consolidação da ESF e como um recurso terapêutico fundamental, o que perpassa uma relação do tipo sujeito-sujeito construída na tessitura das redes de informações existentes no território. O vínculo é constituído, ainda, de um elemento imprescindível para fortalecer as relações no contexto da saúde da família, configurando-se como ferramenta essencial ao cuidado^(36)^.

Estudo multicêntrico realizado com 40 enfermeiras da ESF das cinco regiões do Brasil mostrou que um vínculo positivo com o usuário contribui para facilitar a ação terapêutica, melhorar as respostas aos tratamentos e orientações, e contribuir para reduzir as cargas de trabalho^(37)^. Em consonância, outros pesquisadores^(25)^ apontam que o vínculo é um dos fatores que proporcionam identificação com o contexto da saúde no meio rural e a satisfação com trabalho. Quando mobilizados de forma sensível e situada, esses vínculos constituem linhas de fuga que tensionam o instituído e abrem possibilidades para reinvenções no cuidado.

No campo da formação e prática profissional, os achados deste estudo sugerem que a enfermeira rural pode ser compreendida como cartógrafa do cuidado: alguém que acompanha os processos vivos do território, identifica linhas duras que capturam o trabalho em rotinas repetitivas e detecta linhas de fuga que possibilitam a reinvenção da prática. Essa posição não exige domínio de categorias filosóficas abstratas, mas escuta sensível, atenção às singularidades e disposição para agir em movimento. A cartografia, como abordagem operativa, pode, nesse prisma, ser incorporada na formação das enfermeiras como exercício ético-político de leitura e intervenção territorial, o que estimula análises situadas do processo de trabalho, fortalecendo a autonomia profissional diante das normatizações institucionais.

A partir dessa leitura, destacam-se ainda implicações para gestão do trabalho na APS. A rigidez dos cronogramas, a sobreposição de funções e a centralização do planejamento nas enfermeiras, elementos reconhecidos como linhas duras, apontam para necessidade de estratégias gerenciais que valorizem a escuta ativa das profissionais e promovam maior flexibilidade organizacional. Potencializar espaços de cogestão, incorporar processos participativos de construção das agendas e reconhecer as singularidades dos territórios são ações que podem favorecer práticas mais inventivas e sensíveis. Destarte, os achados do estudo em tela contribuem ainda para repensar modelos de gestão que não apenas administrem recursos, mas que também estimulem vínculos, autonomia e criatividade nos modos de fazer em saúde.

### Limitações do estudo

Além da incipiente literatura de enfermagem rural no Brasil, que restringiu a discussão dos achados, ressalta-se que a realização deste estudo em apenas uma Regional de Saúde pode limitar a transferibilidade dos resultados para outros contextos.

### Contribuições para as áreas da enfermagem e saúde

Compreender as linhas que atravessam os processos de trabalho das enfermeiras rurais oportuniza refletir acerca de formas de aprimoramento da saúde da população rural e das práticas dessas enfermeiras, contribuindo para conformação da enfermagem rural como um campo de saberes e práticas, além de impulsionar o avanço do conhecimento.

## CONSIDERAÇÕES FINAIS

Este estudo mapeou as linhas de força que atravessam os processos de trabalho de enfermeiras rurais, revelando cronogramas rígidos, campanhas verticalizadas e hierarquias institucionais que imobilizam o cuidado, mas também vínculos comunitários que abrem brechas para práticas inventivas e integrais. Esses achados oferecem subsídios para prática profissional ao sugerir agendas flexíveis, espaços de cogestão e formação da enfermeira como cartógrafa do território. Contribuem, ainda, para a formulação de políticas públicas, ao reforçar a necessidade de reconhecer a enfermagem rural como um campo estratégico, com investimento em educação permanente, adequação das cargas de trabalho e financiamento de ações sensíveis às suas singularidades contextuais, o que pode contribuir significativamente para promoção da saúde e do bem-viver das populações rurais.

Sugere-se, ainda, a ampliação do uso da cartografia para diferentes contextos regionais, explorando suas potencialidades analíticas e interventivas. Estudos que articulem métodos qualitativos e quantitativos podem contribuir para avaliar os efeitos dessas possíveis intervenções sobre o vínculo com a comunidade, a qualidade do cuidado e a valorização profissional, fortalecendo a cartografia como ferramenta de gestão e planejamento em saúde, e contribuindo para políticas públicas mais sensíveis às realidades locais.

## Data Availability

Os dados de pesquisa estão disponíveis no corpo do artigo.
